# *IDH* Mutations: Genotype-Phenotype Correlation and Prognostic Impact

**DOI:** 10.1155/2014/540236

**Published:** 2014-04-30

**Authors:** Xiao-Wei Wang, Pietro Ciccarino, Marta Rossetto, Blandine Boisselier, Yannick Marie, Virginie Desestret, Vincent Gleize, Karima Mokhtari, Marc Sanson, Marianne Labussière

**Affiliations:** ^1^Université Pierre et Marie Curie-Paris 6, Centre de Recherche de l'Institut du Cerveau et de la Moëlle épinière (CRICM) UMR-S975, 75013 Paris, France; ^2^INSERM U 975, 75013 Paris, France; ^3^CNRS, UMR 7225, 75013 Paris, France; ^4^Institut du Cerveau et de la Moëlle épinière (ICM), Plateforme de Génotypage Séquençage, 75013 Paris, France; ^5^AP-HP, Groupe Hospitalier Pitié-Salpêtrière, Laboratoire de Neuropathologie R. Escourolle, 75013 Paris, France; ^6^AP-HP, Groupe Hospitalier Pitié-Salpêtrière, Service de Neurologie 2, 75013 Paris, France; ^7^Fédération de Neurologie Mazarin, Groupe Hospitalier Pitié-Salpêtrière, 75651 Paris Cedex 13, France

## Abstract

*IDH1/2* mutation is the most frequent genomic alteration found in gliomas, affecting 40% of these tumors and is one of the earliest alterations occurring in gliomagenesis. We investigated a series of 1305 gliomas and showed that *IDH* mutation is almost constant in 1p19q codeleted tumors. We found that the distribution of *IDH1^R132H^*, *IDH1^nonR132H^*, and *IDH2* mutations differed between astrocytic, mixed, and oligodendroglial tumors, with an overrepresentation of *IDH2* mutations in oligodendroglial phenotype and an overrepresentation of *IDH1^nonR132H^* in astrocytic tumors. We stratified grade II and grade III gliomas according to the codeletion of 1p19q and *IDH* mutation to define three distinct prognostic subgroups: 1p19q and *IDH* mutated, *IDH* mutated—which contains mostly *TP53* mutated tumors, and none of these alterations. We confirmed that *IDH* mutation with a hazard ratio = 0.358 is an independent prognostic factor of good outcome. These data refine current knowledge on *IDH* mutation prognostic impact and genotype-phenotype associations.

## 1. Introduction 

The WHO Classification of Tumors of the Central Nervous System is the universal standard for classifying and grading brain neoplasms [1]. According to the presumed cell of origin, gliomas have been classified into three major groups: astrocytomas, oligodendrogliomas, and mixed oligoastrocytomas. Based on the presence or absence of malignant features: cell density, nuclear atypia, mitosis, microvascular proliferation, and necrosis, the WHO classification distinguishes grades I, II (LGG), III (anaplastic), and IV (glioblastomas, GBM) [2]. However, this classification suffers from a lack of reproducibility, with a high interobserver variability, often leading to discordant results between centers [3–5].

In these settings, there is a need for the identification of additional prognostic markers to refine the WHO classification in order to define more homogeneous subgroups. Mutations in the* IDH1* (isocitrate dehydrogenase 1) gene have been first reported in 2008 [6]. Since then, the* IDH1* mutation has been recognized as the most frequent alterations in gliomas, occurring in 40% of glial tumors [7–9] and is the most powerful prognostic factor ever described in gliomas [10, 11]. Less frequently the mitochondrial isoform* IDH2* is mutated.

We have investigated the mutational status of* IDH1* and* IDH2* in a cohort of 1305 glioma patients and correlated it with the genomic profile and the outcome.

## 2. Patients and Methods

### 2.1. Patients and Tissue Samples

Patients were selected retrospectively according to the following criteria: histologic diagnosis of grade II to grade IV glioma; clinical data and follow-up available in the neurooncology database; and written informed consent. The inclusion period extends from May 1987 to October 2010. Tumor DNA was extracted from both frozen and paraffin embedded formalin fixed tumors, when available, using the QIAmp DNA minikit, as described by the manufacturer (Qiagen). CGH-array analysis, LOH (loss of heterozygosity) analysis,* EGFR* amplification, and* P16* deletion assessment were performed as previously described [12].

### 2.2. Determination of* IDH1* and* IDH2* Mutational Status

The genomic regions spanning wild-type R132 of* IDH1* and wild-type R172 of* IDH2* were analyzed by direct sequencing using the following primers: IDH1f 5-AGAAGAGGGTTGAGGAGTTCAA, IDH1r 5-CACATACAAGTTGGAAATTTCTGG, IDH2f 5-AGCCCATCATCTGCAAAAAC, and IDH2r 5-CTAGGCGAGGAGCTCCAGT, as previously described [10]. Forward and reverse chains were analyzed on an ABI prism 3730 DNA analyzer (Perkin Elmer).

*IDH2* mutational status was determined by Sanger sequencing and by PCR HRM. The latter approach allowing only the detection of an* IDH2* mutation presence, we have only the type of base substitution for 15 tumors. HRM was performed as previously described [13].

### 2.3. *MGMT* Status and* TP53* Mutations Determination

DNA methylation status of the* MGMT* promoter was determined by bisulfite modification and subsequent nested MSP, a two-stage PCR approach, as previously described [14].

*TP53* gene mutations were screened for exons 5–8 by using previously reported primers and methods [15].

### 2.4. Statistical Analysis

The *χ* ^2^ test (or Fisher's exact test when one subgroup was <5) was used to compare the genotype distribution. The association with continuous variables was calculated with a Mann-Whitney test.

Overall survival (OS) was defined as the time between the diagnosis and death or last follow-up. Patients who were still alive at last follow-up were considered as a censored event in analysis. Progression free survival (PFS) was defined as the time between the diagnosis and recurrence or last follow-up. Patients who were recurrence-free at last follow-up were considered as a censored event in analysis. To find clinical and/or genomic factors related to OS (or PFS), survival curves were calculated according to the Kaplan-Meier method and differences between curves were assessed using the log-rank test. Variables with a significant *P* value were used to build multivariate Cox model.

## 3. Results

We have screened for the presence of codon-132 mutations in the* IDH1* gene in a large cohort of 1305 gliomas, including 436 WHO grade II, 394 WHO grade III, and 475 WHO grade IV gliomas. The presence of* IDH2* mutation was investigated in a cohort of 980 gliomas (379 grade II, 289 grade III, 312 grade IV). In the whole cohort, sex ratio was 1.3 and median age at diagnosis was 49.2 years (range, 16.1 to 89.1 years). The characteristics of the population are indicated in Table 1.

Taken together we found 609/1305* IDH1* and 30/980* IDH2* mutations (global mutation rates of 46.7% and 3.1%, resp.). No tumor harbored both* IDH1* and* IDH2* mutations (Supplementary Table 1 available online at http://dx.doi.org/10.1155/2014/540236). Patients with IDH1 mutations were younger for the whole series (median age 40.6 years for IDH1 mutated patients versus 55.9 years; *P* < 0.0001) and also for grades III and IV separately (median age at diagnosis 44.4 and 47.8 years for grades III and IV* IDH* mutated tumors, versus 51.5 and 59.0 years for grades III and IV nonmutated gliomas; *P* = 0.0012 and *P* < 0.0001, resp.).

### 3.1. Genotype-Phenotype Correlations

*IDH1* mutations affected 72.5% (316/436) grade II, 63.7% (251/394) grade III, and 8.8% (42/475) grade IV gliomas. We looked then for association between glioma subtypes (astrocytic, mixed, and oligodendroglial tumors) and* IDH1* ^*R132H*^,* IDH1* ^*nonR132H*^ mutations, and* IDH2* mutations. In grades II and III gliomas,* IDH2* mutations were overrepresented in oligodendrogliomas (22 IDH2 mutations out of 330* IDH* mutated tumors; 6.7%), compared to astrocytomas (1/60; 1.7%) and mixed gliomas (6/176; 3.4%) (*P* = 0.049). In contrast, we found that* IDH1* ^*nonR132H*^ mutations were more frequent in astrocytic (6/60, 10.0%* IDH* mutated tumors) and mixed tumors (15/176, 8.5%), compared to oligodendroglial tumors (15/332, 4.5%, *P* = 0.037).

### 3.2. *IDH* Mutations Are Associated with Tumor Genomic Profile

We have then evaluated the association of* IDH* mutation with the molecular alterations commonly found in gliomas (Table 2). We found that* IDH* mutations were significantly associated with* MGMT* promoter methylation (*P* < 0.0001). In contrast, there was a strong association between the absence of* IDH* mutation and complete loss of chromosome 10q,* EGFR* amplification and* P16* deletion (*P* < 0.0001 in each case).

Complete 1p19q codeletion was found in 150 gliomas: the* IDH1* gene was mutated in 137 cases (91.3%) and the* IDH2* gene was mutated in 12 of the 13 remaining tumors. Taken together, the* IDH* genes were altered in 99.3% (149/150) of the 1p19q codeleted tumors.

*TP53* mutation was analyzed by Sanger sequencing in 396 tumors: 64/178 (35.9%)* IDH* mutated tumors were also mutated on* TP53*, versus 49/218 (22.5%) of the nonmutated tumors (*P* = 0.0036).* TP53* mutation was correlated with astrocytic histology: 95 tumors out of 286 (33.2%) astrocytic and mixed gliomas were* TP53* mutated, whereas only 16.4% (18/110) of oligodendrogliomas were mutated on* TP53* (*P* = 0.0008).* TP53* mutation was rarely associated with 1p19q codeletion: 1p19q codeleted gliomas were less frequently* P53* mutated (4/52, 7.7%), as compared to the noncodeleted tumors (103/170, 60.6%; *P* < 0.0001). When excluding 1p19q codeleted tumors (considered as the hallmark of oligodendrogliomas),* TP53* mutation was even more strongly correlated with IDH mutation: 57/98 (58.2%) of* IDH* mutated tumors was also mutated on* TP53*, versus 46/175 (26.2%, *P* < 0.0001) in nonmutated gliomas.

### 3.3. *IDH1* Mutation Is an Independent Prognostic Factor of Good Outcome

**Figure 1:**
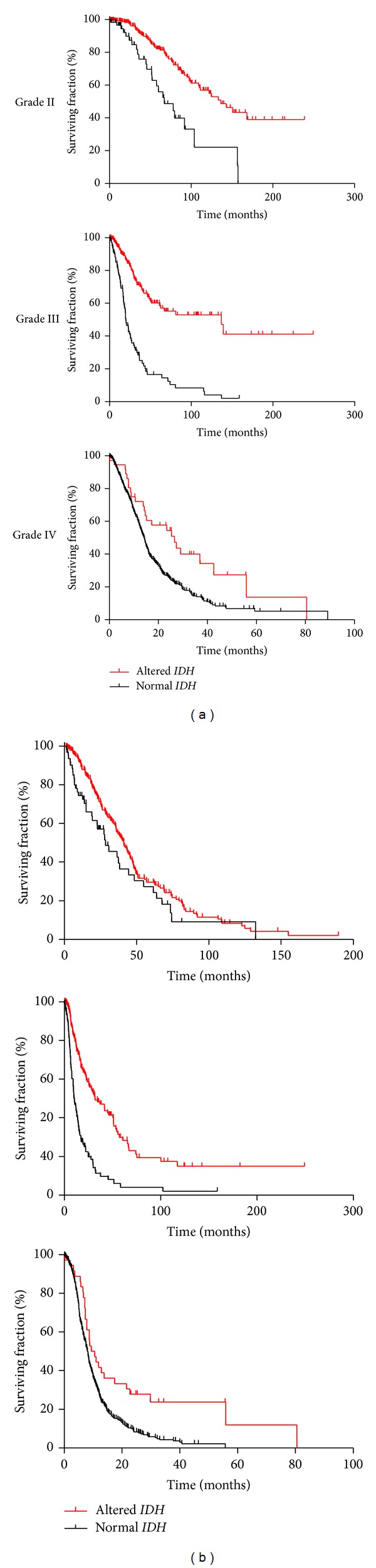
Prognostic impact of* IDH* status on overall survival (a) and progression free survival (b) in grade II to IV gliomas.

We investigated the prognostic impact of* IDH* status in grade II, grade III, and grade IV gliomas. For each grade,* IDH* mutated patients have significantly longer overall survival and progression free survival than* IDH* normal patients (Figure 1 and Table 2).

We then entered the following factors as candidate variables in the multivariate Cox proportional hazards regression model analysis:* IDH* mutation,* P16* deletion, 1p19q codeletion, extent of surgery, Karnofsky index, and age at diagnosis (Table 3).* IDH* mutation was a strong and independent predictor of a better outcome (hazard ratio for overall survival= 0.358; 95% CI, 0.248 to 0.517; *P* < 0.0001).

**Figure 2:**
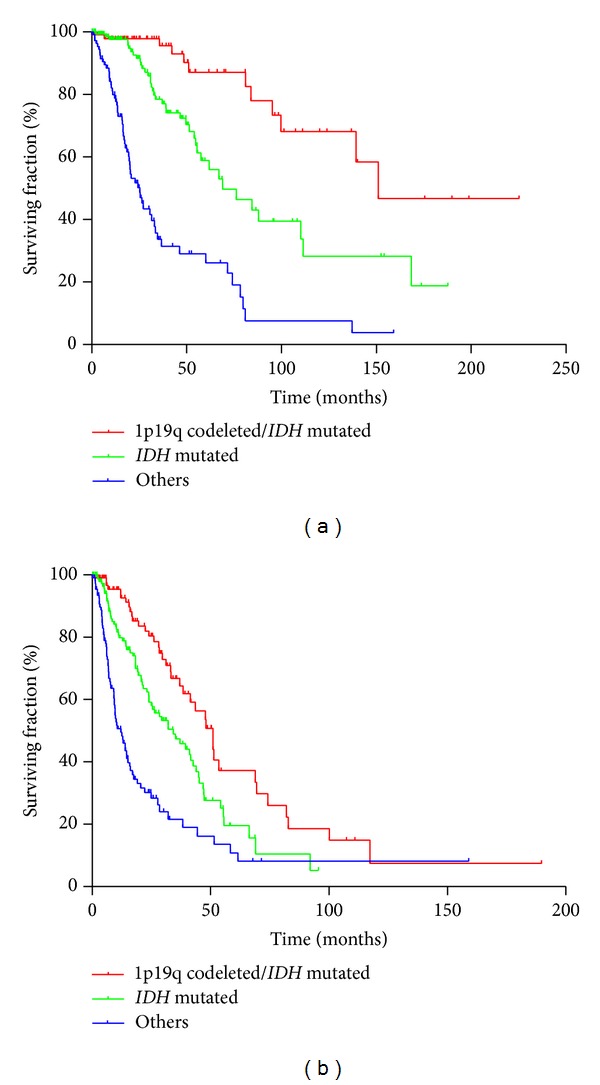
Overall survival (OS, (a)) and progression free survival (PFS, (b)) for grade II and III gliomas patients stratified according to 1p19q codeletion and presence of IDH mutations. Median OS were 150.9, 69.0, and 25.4 months for 1p19q/IDH mutated, IDH mutated, and other groups, respectively. Median PFS were 51.1, 34.3, and 12.2 months for 1p19q/IDH mutated, IDH mutated, and other groups, respectively.

Moreover, as previously described [16], we stratified the grade II and grade III tumors according to 1p19q codeletion and* IDH* status, thus defining three prognostic groups: 1p19q codeleted (and* IDH* mutated),* IDH* mutated, and others (Figure 2).

Whatever the grade, patients harboring the 1p19q codeletion have a significantly longer survival (median OS: 150.9 months) than patients only harboring* IDH* mutation (69.0 months) or none of these alterations (25.4 months). We looked then at* TP53* mutation in these three prognostic groups and found* P53* mutation strongly associated with group 2 in both grades II and III (Table 4). For example in grade II gliomas,* TP53* was mutated in 58.5% in group 2, versus 8.8% and 27.8% in groups 1 and 3, respectively (*P* < 0.0001 and *P* = 0.031, resp.).

## 4. Discussion

In this large series, we investigated the place of* IDH1*/*IDH2* mutation in gliomas, in particular in different genotypes and phenotypes. As a first result, we confirmed the strong association of* IDH* mutations with the tumor genomic profile [10]: virtually all 1p19q codeleted tumors are* IDH* mutated [17, 18] whereas* IDH* mutation is extremely rare in gliomas with* EGFR* amplification. Secondly, we showed that the type of mutation is related to the molecular profile. The* IDH1* ^*R132H*^ mutation represents 90% of all* IDH* mutations. However, we found here that* IDH1* ^*nonR132H*^ mutations are associated with astrocytic tumors [19], whereas* IDH2* mutations are associated with oligodendrogliomas. The 1p19q codeletion is a hallmark of oligodendroglial phenotype and we found similar results when tumors are stratified according to histological subtype.

The association of* IDH* mutation with* TP53* mutation has been widely studied in literature and has led to contradictory results.* IDH* mutation was found associated with* TP53* mutation in several studies [11, 18, 20–24] but other authors did not find such an association [10, 25]. We found an association between* IDH* and* TP53* mutations, but we showed* TP53* mutation correlated with astrocytic phenotype, in contrast with* IDH* mutation more associated with the oligodendroglial phenotype. Therefore, when excluding 1p19q codeleted tumors, mostly oligodendroglial, and rarely* TP53* mutated, we found a stronger positive association between* IDH* and* TP53* mutations. This result is concordant with the data of Gravendeel et al. who found a correlation between* TP53* mutation and *IDH*1^*nonR*132*H*^ mutation [26].

Confirming previous data obtained on smaller cohorts [10, 16], our findings showed that gliomas patients harboring an* IDH1* mutated tumor present an improved outcome, compared to patients with an* IDH1* normal tumor. The multivariate analysis shows that* IDH* status is an independent prognostic factor in a 1332 glioma patients cohort. To further explore the prognostic impact of* IDH1* mutation, we subdivided both grade II and III gliomas patients in three prognostic subgroups, based on the 1p19q codeletion and* IDH1* mutation status ((i)* IDH* mut/1p19qdel, (ii)* IDH* mut/1p19qnon del, (iii)* IDH* non mut/1p19qnon del.). In line with a recent study [22], we found that* TP53* mutation characterizes the group 2 (IDH mut non 1p19q codeleted). The third group with the worst prognosis contains mainly triple negative gliomas (non 1p19q codeleted, non* IDH* mutated, non* TP53* mutated) [22].

Taken together, our results show that* IDH* mutation combined with other genomic marker can be used to refine the prognostic classification of gliomas, independently of tumor grade. With the recent results of randomized trial,* IDH1* mutation has become, with 1p19q codeletion, a predictive marker of the response to chemotherapy [27–29].

## Supplementary Material

Supplementary Table 1: Describes the frequency of the different types of IDH1 and IDH2 mutations found in the 1305 glioma patients cohort.

## Figures and Tables

**Table 1:** Patients demographics and clinical characteristics.

	Glioma by grade		
Characteristics	II (*n* = 436)	III (*n* = 394)	IV (*n* = 475)
Age, years			
Median	38.1	47.8	58.5
Range	16.1–77.0	19.1–89.1	18.2–89.1
KPS			
Median	90	90	80
Range	50–100	60–100	40–100
Biopsy (%)	25.6	28.7	26.6
Tumor removal (%)	74.4	71.3	73.4
Overall survival, months			
Median	121.9	41.7	14.5
Range	0.1–238.9	0.1–249.3	0.1–89.1
Progression free survival, months			
Median	38.8	19.5	8.2
Range	0.1–189.7	0.1–249.3	0.1–80.5

KPS: Karnofsky performance score; PFS: progression-free survival.

**Table 2:** Comparison of histologic distribution, molecular alterations, and prognostic impact between *IDH* mutated and wild type patients.

		*n*	*IDH1* mutated tumors*	*IDH2* mutated tumors	*IDH *wild type tumors
Histologic subtypes	Astrocytic tumors	**448**	**87**	**2**	**359**
	AII	61	43 (2)	1	17
	AIII	33	17 (4)	0	16
	GBM	354	27 (1)	1	326
	Oligodendroglial tumors	**584**	**347**	**22**	**215**
	OII	243	182 (10)	15	46
	OIII	220	150 (5)	7	63
	GBMO	121	15 (1)	0	106
	Mixed tumors	**275**	**176**	**6**	**93**
	OAII	134	92 (6)	5	37
	OAIII	141	84 (9)	1	56

Molecular alterations	*MGMT* promoter methylation	587	195/256 (76.2%)		172/331 (52.0%)
	*EGFR* amplification	1248	9/609 (1.5%)		196/639 (30.7%)
	Complete 10q loss	1148	57/572 (10.0%)		359/576 (62.3%)
	*P16* deletion	1232	63/595 (10.6%)		203/637 (31.8%)
	*TP53* mutation	396	64/178 (35.9%)		49/218 (22.5%)

Prognostic impact	Overall survival				
	Grade II	309	136.5		67.0^a^
	Grade III	303	136.9		20.1^b^
	Grade IV	435	26.6		14.2^c^
	Progression free survival				
	Grade II	309	41.3		28.5^d^
	Grade III	303	31.9		10.4^e^
	Grade IV	435	10.0		8.1^f^

*For histologic subtypes, the number in parentheses indicates the number of *IDH*1^*nonR*132*H*^ mutations. ^a,b,e^ *P* < 0.0001; ^c^ *P* = 0.0004; ^d^ *P* = 0.0363; ^f^ *P* = 0.0008.

**Table 3:** Multivariate Cox proportional hazards regression model analysis of survival of the 1305 glioma patients cohort. *MGMT* promoter methylation was not included in this analysis due to a low number of evaluable patients for this parameter.

Parameter	Overall survival			Progression free survival		
	HR	95% CI for HR	*P*	HR	95% CI for HR	*P*
Age > 60 years	1.831	1.358 to 2.467	0.0001	1.479	1.158 to 1.889	0.0018
Surgery extent	0.775	0.588 to 1.021	0.0715	1.045	0.823 to 1.326	0.7199
1p19q codeletion	0.202	0.098 to 0.415	<0.0001	0.491	0.326 to 0.739	0.0007
*IDH* mutation	0.358	0.248 to 0.517	<0.0001	0.467	0.348 to 0.627	<0.0001
IK > 70	0.419	0.315 to 0.556	<0.0001	0.489	0.375 to 0.636	<0.0001
*P16* deletion	1.513	1.168 to 1.960	0.0018	1.471	1.165 to 1.858	0.0013

**Table 4:** Association of *TP53* mutation with 1p19q codeleted tumors and *IDH* mutated tumors.

		*TP53 *			
		Mutated	Normal	Percentage	Difference to *IDH* mutated group (*P*)
Grade II	1p19q/*IDH* mutated	3	31	8.8%	*<0.0001 *
	*IDH* mutated	31	22	58.5%	*— *
	others	5	13	27.8%	*0.0309 *

Grade III	1p19q/*IDH* mutated	1	16	6.3%	*0.0002 *
	*IDH* mutated	21	13	61.8%	*— *
	others	11	24	31.4%	*0.0160 *
