# Supplementary material for: IDH Mutations: Genotype-Phenotype Correlation and Prognostic Impact
Source: Biomed Res Int. 2014 Apr 30;2014:540236. doi: 10.1155/2014/540236 (PMC4022066; doi:10.1155/2014/540236)
Supplement: Supplementary file 1 — Supplementary Table 1: Describes the frequency of the different types of IDH1 and IDH2 mutations found in the 1305 glioma patients cohort. [file 540236.f1.pdf]

SUPPLEMENTARY TABLES

Supplementary Table I

	Nucleotide change	Amino acid change	N (%)
IDH1			**609/1305 (46.7%)**
	G395A (CAT)	Arg132His	571/609 (93.8%)
	C394T (TGT)	Arg132Cys	15/609 (2.5%)
	C394G (GGT)	Arg132Gly	12/609 (2.0%)
	C394A (AGT)	Arg132Ser	7/609 (1.1%)
	G395T (CTT)	Arg132Leu	4/609 (0.6%)
IDH2			**30/980 (3.1%)**
	G515A (AAG)	Arg172Lys	11/15 (73.3%)
	G516T (AGT)	Arg172Ser	3/15 (20.0%)
	G515T (ATG)	Arg172Met	1/15 (6.7%)
